# Therapeutic effect of adipose-derived mesenchymal stem cells in a porcine model of abdominal sepsis

**DOI:** 10.1186/s13287-023-03588-x

**Published:** 2023-12-12

**Authors:** J. F. Vélez-Pinto, M. Garcia-Arranz, D. García-Bernal, S. García Gómez-Heras, P. Villarejo-Campos, A. M. García-Hernández, L. Vega-Clemente, S. Jiménez-Galanes, H. Guadalajara, J. M. Moraleda, D. García-Olmo

**Affiliations:** 1 Surgery Department, Fundación Jiménez Díaz University Hospital, 28033 Madrid, Spain; 2 New Therapy Laboratory, Health Research Institute of the Jimenez Diaz Foundation (Instituto de Investigacion Sanitaria de la Fundacion Jimenez Diaz), Avda Reyes Católicos 2, 28040 Madrid, Spain; 3 Department of Surgery, Faculty of Medicine, Universidad Autónoma de Madrid, 28029 Madrid, Spain; 4 Hematopoietic Transplant and Cellular Therapy Unit, Instituto Murciano de Investigación Biosanitaria (IMIB) Pascual Parrilla, Virgen de la Arrixaca University Hospital, University of Murcia, Murcia, Spain; 5 Biochemistry, Molecular Biology and Immunology Department, Faculty of Medicine, University of Murcia, Murcia, Spain; 6 Department of Basic Health Science, Faculty of Health Sciences, Rey Juan Carlos University, 28922 Alcorcón, Madrid, Spain; 7 Department of Surgery, Infanta Elena University Hospital, 28342 Valdemoro, Madrid, Spain

**Keywords:** Peritoneal sepsis, Adipose-derived mesenchymal stem cells, Xenogeneic use, Peritoneal injection

## Abstract

**Background:**

The term sepsis refers to a complex and heterogeneous syndrome. Although great progress has been made in improving the diagnosis and treatment of this condition, it continues to have a huge impact on morbidity and mortality worldwide. Mesenchymal stem cells are a population of multipotent cells that have immunomodulatory properties, anti-apoptotic effects, and antimicrobial activity. We studied these capacities in a porcine model of peritoneal sepsis.

**Methods:**

We infused human adipose-derived mesenchymal stem cells (ADSCs) into a porcine model of peritoneal sepsis. Twenty piglets were treated with antibiotics alone (control group) or antibiotics plus peritoneal infusion of ADSCs at a concentration of 2 × 10^6^ cells/kg or 4 × 10^6^ cells/kg (low- and high-dose experimental groups, respectively). The animals were evaluated at different time points to determine their clinical status, biochemical and hematologic parameters, presence of inflammatory cytokines and chemokines in blood and peritoneal fluid, and finally by histologic analysis of the organs of the peritoneal cavity.

**Results:**

One day after sepsis induction, all animals presented peritonitis with bacterial infection as well as elevated C-reactive protein, haptoglobin, IL-1Ra, IL-6, and IL-1b. Xenogeneic ADSC infusion did not elicit an immune response, and peritoneal administration of the treatment was safe and feasible. One day after infusion, the two experimental groups showed a superior physical condition (e.g., mobility, feeding) and a significant increase of IL-10 and TGF-β in blood and a decrease of IL-1Ra, IL-1b, and IL-6. After 7 days, all animals treated with ADSCs had better results concerning blood biomarkers, and histopathological analysis revealed a lower degree of inflammatory cell infiltration of the organs of the peritoneal cavity.

**Conclusions:**

Intraperitoneal administration of ADSCs as an adjuvant therapy for sepsis improves the outcome and diminishes the effects of peritonitis and associated organ damage by regulating the immune system and reducing intra-abdominal adhesions in a clinically relevant porcine model of abdominal sepsis.

**Supplementary Information:**

The online version contains supplementary material available at 10.1186/s13287-023-03588-x.

## Introduction

Sepsis refers to a complex and heterogeneous syndrome. In 2014, the Third International Consensus (Sepsis-3) defined sepsis and septic shock as organ dysfunction due to a dysregulated host response to infection [1]. Although great improvements have been made in the diagnosis and treatment of sepsis, it continues to have a substantial impact on morbidity and mortality worldwide. There are approximately 49 million cases of sepsis per year globally, resulting in 11 million deaths, or 19.7% of all deaths in the world [2]. In the clinical setting, the Sequential Organ Failure Assessment (SOFA) score is used to evaluate organ dysfunction. In the present study, we used a pig-specific SOFA (pSOFA) scale (Table 1), which draws on a series of clinical and laboratory findings to determine syndrome severity and the efficacy of treatment [3].

**Table 1:** Pig-specific sequential organ function scoring system

Organ dysfunction	Parameters	Score value				
		0	1	2	3	4
Respiration	PaO_2_/FiO_2_ ratio	> 400	< 400	< 300	< 200	< 100
Cardiovascular	MAP (mmHg)	> 75	< 75	< 65	N < 0.1	N > 0.1
Renal	Urine output (mLkg^−1^ h^−1^)	> 0.5	< 0.5	< 0.25	F < 10	F > 10
Liver	Bilirubin (µmol L^−1^)	< 20	> 20	> 32	> 101	> 204
Coagulation	Platelet count (× 10^9^ L^−1^)	> 200	< 200	< 150	< 100	< 50

pSOFA. Adaptation of the Sequential Organ Function Assessment scoring system to evaluate sepsis-induced organ dysfunction in pigs

*PaO*_*2*_ arterial partial pressure of oxygen dissolved in plasma, *FiO*_*2*_ fraction of inspired oxygen, *MAP* mean arterial pressure, *N* norepinephrine, *F* furosemide

Extracted from Rutai et al. [3]

The abdomen is the second most common site of infection in patients with sepsis after the lungs [4]. Abdominal sepsis can have a variety of causes including primary or secondary peritonitis, postoperative complications, etc. The mortality rate of complicated intra-abdominal infection is 9.2% [5].

Initial treatment of sepsis consists of fluid resuscitation, broad-spectrum antibiotics, and source control. Even with appropriate management, however, morbidity and mortality remain unacceptably high and new therapeutic strategies are needed [6]. One such emerging strategy is stem-cell therapy.

Mesenchymal stem cells (MSCs) are a population of multipotent non-hematopoietic stem cells capable of differentiating into a variety of cell types including adipocytes, myocytes, endothelial cells, osteoblasts, and other mesoderm-derived cells [7]. MSCs have immunomodulatory properties [8], anti-apoptotic effects, and antimicrobial activity [9–11] and have shown positive preclinical results in animal models of sepsis, reducing mortality, although large-animal studies are needed [12].

MSCs can be isolated from different tissues, including bone marrow, adipose tissue, Wharton’s jelly, peripheral blood and dental pulp, among others. For surgeons, adipose tissue is the most convenient source of MSC, and adipose-derived MSCs have demonstrated good efficacy in treating peritoneal sepsis in murine preclinical models [13, 14]. In addition, allogeneic MSCs from healthy donors might be used, since these cells do not express MHC class II molecules, and the activity of MHC class I molecules is very low [15]. Therefore, numerous studies have used xenogeneic MSCs, taking advantage of this property that makes them immunoprivileged cells [16].

A previous study in a porcine model of peritoneal sepsis that used intravenously delivered allogeneic bone marrow MSCs reported no treatment effect [17]. In the present study, we made three relevant modifications to this earlier research: We used xenogeneic cells, adipose-derived MSCs, and performed intraperitoneal infusion. This study evaluates the effects of these modifications in a clinically relevant porcine model of peritonitis.

## Methods

### Isolation and characterization of adipose-derived MSCs

After informed consent was provided, abdominal fat from healthy human donors was aspirated via liposuction. Under good manufacturing practice (GMP) conditions, the lipoaspirate was first centrifuged to separate the adipose tissue from other components. It was then digested in collagenase type I (1 mg/mL) (Nordmark Pharma GmbH, Uetersen, Germany) under gentle agitation for 45 min at 37 °C. The digested tissue was sequentially filtered with Dulbecco´s phospate-buffered saline (DPBS) and washed with PBS (phosphate-bufferred saline, Gibco, Invitrogen Corporation, Waltham, MA, USA) to remove cellular debris. The pellet was then resuspended in Dulbecco's modified Eagle's medium (Gibco) and seeded at a density of 5 × 10^3^ cells/cm^2^ under standard culture conditions (37 °C in a humidified atmosphere containing 5% CO_2_). Growth media supplemented with 5% fetal bovine serum (FBS, Gibco), FGF-β (Biotechne, Minnesota,USA), and 1% gentamycin (Normon, Madrid, Spain) were changed every 3–4 days. When the cells reached 70–80% confluence, they were detached with TrypLE Select enzyme (Gibco) and subcultured at a density of 5 × 10^3^ cells/cm^2^. A cell stock of ADSCs resuspended in HypoThermosol FRS preservation medium (Merck KGaA, Darmstadt,Germany) supplemented with 10% dimethyl sulfoxide (DMSO) or in Cryostor CS10 medium (Merck KGaA, Germany) was cryopreserved on second passage. The cells were stored in liquid nitrogen until use.

When required, bags of cryopreserved cell stock were thawed at 37 °C, washed with DPBS to remove the cryopreservant agent DMSO and seeded at 5–8 × 10^3^ cells/cm^2^ to confluence. The final product consisted of vials containing 50 × 10^6^ of ADSC resuspended in Ringer’s lactate (Grifols, Spain) and 1% human serum albumin (Grifols, Barcelona, Spain) at a concentration of 2 × 10^6^ cells/ml and 4 × 10^6^ cells/ml that were shipped at 2–8 °C.

Characterization of the final product consisted of the following: cell viability assessed by trypan blue staining (Sigma-Aldrich, St. Louis, MO, USA); analysis of expression of CD73, CD90, CD44, CD34, and CD45 antigen by flow cytometry; and measurement of immunomodulatory potency by lymphocyte proliferation assay in co-culture with ADSCs. Microbiological controls included mycoplasma detection (VenorGeM OneStep Mycoplasma, Minerva Labs, Petah Tikva, Israel), sterility testing (BacT/ALERT 3D system; bioMerieux, Marcy-l'Étoile, France), detection of adventitious viruses by observation of cytopathic effect in cell cultures, and endotoxin levels (Endosafe PTS, Charles River Laboratories, Wilmington, MA, USA).

### Experimental animals

Twenty crossbred Landrace-Large White piglets of either sex, weighing between 25 and 35 kg, were obtained from a conventional breeding facility. The animals were housed in separate pens for the duration of the experiments. The contents of EU Directive 2010/63 on the protection of animals used for scientific purposes were upheld at all times, as were Spanish regulations (RD 53/20013). The study was reviewed and approved by the Ethics Committee of the Community of Madrid for animal experimentation (No-PROEX 235/19) on January 17, 2020, with the title: Treatment of peritoneal sepsis with adipose-derived mesenchymal stem cells in a porcine model. All experiments involving animals were carried out at the Instituto de Investigación Sanitaria Fundación Jimenez Diaz. The research team supervised activities concerning animal control and welfare in collaboration with the staff of the Fundación Jiménez Díaz University Hospital.

### Experimental protocol

Twenty animals were assigned to three experimental groups in open-labeled fashion: (1) Control group: *n* = 5 animals, receiving standard sepsis treatment (i.e., iv fluids, antibiotics, and source control); (2) low-dose ADSC to treat sepsis: *n* = 10 animals, with standard treatment plus abdominal compartment syndrome (ACS) treatment at a dose of 2 × 10^6^/kg; and (3) high-dose ADSC to treat sepsis: *n* = 5 animals, with standard treatment plus ACS treatment at a dose of 4 × 10^6^/kg. The animals arrived at the facility at least 48 h prior to the first procedure and fasted for 24 h before surgery.

**Fig. 1:**
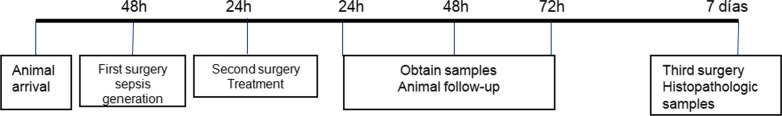
Study flowchart

All animals underwent three surgeries (Fig. 1, Flowchart): induction of peritoneal sepsis, treatment of sepsis at 24 h, and evaluation of the disease course 7 days after treatment. All surgeries followed the same anesthetic protocol: The animals were premedicated with a combination of intramuscular ketamine (8 mg/kg), midazolam (0.6 mg/kg), xylazine (2.2 mg/kg), and atropine (0.01 mg/kg). Once adequate sedation was achieved, the pigs were intubated using an endotracheal tube with an internal diameter of between 6.5 and 7.5 mm. During the operation, we monitored the results of an electrocardiogram (ECG), pulse oximeter (SpO_2_), capnography (EtCO_2_), and anesthetic gases (EtIso); rectal temperature was also recorded. A central venous catheter was placed via the external jugular vein and tunneled to the back of the neck to facilitate blood collection and medication infusion over the subsequent days.

#### First procedure

Induction of fecal peritonitis using a porcine model adapted from Kubiak et al. [18]. With the animal under anesthesia, a small midline laparotomy was performed, intraabdominal temperature was measured, the cecum was identified, and a 2-cm enterotomy was performed. Subsequently, 0.5 mL/kg of feces was obtained and the enterotomy was closed with an absorbable 3/0 running monofilament suture. The feces were mixed with 2 mL/kg of blood and left to stand for 15 min until a clot formed. The clot was then distributed in the left lower quadrant, and the laparotomy was closed. The animal was then awakened and returned to the pen for recovery. The animal was given water but no food. During surgery, 1 L of Ringer’s lactate was infused intravenously. Antibiotic treatment was initiated, consisting of a single dose of ceftriaxone 1 g and metronidazole 1500 mg every 24 h and continuing for 4 days as of the onset of fecal peritonitis. For analgesia, an intravenous injection of 15 mg of meloxicam and 25 mg of tramadol was infused during the procedure and repeated daily. A transdermal patch containing 12.5 μg/h fentanyl was applied and kept in place for 72 h.

#### Second procedure

Treatment. After 24 h, the animals were anesthetized and the laparotomy was reopened and extended. The intra-abdominal temperature was measured, the abdomen was examined and findings recorded, the cecum was located, and the enterotomy was checked for suture dehiscence. Gross contamination was removed, and abdominal lavage was performed with 6 L of warm saline. After the first 3 L of lavage, peritoneal lavage fluid was collected for cytokine analysis. Following abdominal lavage, intraperitoneal instillation of the saline solution or ADSCs was applied, depending on the study group, distributing the dose throughout the abdominal cavity. The laparotomy was closed, and the animal was then awakened and placed in the pen for recovery. Analgesia and antibiotic treatment were maintained.

#### Third procedure

Kill 7 days after the first procedure. Animals were anesthetized, the laparotomy was reopened, all tissues (small intestine, peritoneum, spleen, kidney, and liver) were collected for histopathological examination, and biological samples were collected. The animals were then euthanized by intravenous barbiturate and potassium overdose.

#### Follow-up

Animals were examined, and the findings and behavior were recorded according to the protocol approved by the animal welfare committee. Analgesia, antibiotics, and 500 cc of Ringer’s lactate were infused, and rectal temperature was recorded daily. Water and food were provided ad libitum.

#### Sample collection

Peritoneal lavage fluid was obtained during the first and second surgeries for analysis of pro-inflammatory and anti-inflammatory molecules as well as acute phase reactants. Blood samples were collected in all surgeries, i.e., 24, 48, and 72 h after treatment. Plasma was subsequently obtained by blood centrifugation at 1800 × *g* for 10 min at 4 °C and a second centrifugation at 3000 × *g* for 10 min at 4 °C. Blood and peritoneal lavage samples were collected for microbiological studies at the beginning of the first and second surgeries.

Blood samples were collected to determine baseline complete blood count, partial thromboplastin time, prothrombin time, international normalized ratio (INR), ferritin, fibrinogen, glucose, creatinine, bilirubin, alanine aminotransaminase (ALT), gamma-glutamyl transferase (GGT), lactate dehydrogenase (LDH), and cytokine analysis. For the latter, blood and peritoneal lavage were centrifuged at 1000 × *g* for 15 min at 4 °C and a second centrifugation at 10 000 × *g* for 15 min at 4 °C. Analysis of pro-inflammatory cytokines and chemokines in blood plasma and peritoneal lavage was performed by immunoassay using the Plex PCYTMAG23PMX13BK by Millipore (Porcine Cytokine/Chemokine Magnetic Bead Panel Space Saver Packaging), which determines the following biomarkers: GM-CSF, IFNγ, IL-1a, IL-1b, IL 1Ra, IL-2, IL-4, IL-6, IL-8/CXCL8, IL-10, IL-12, IL-18, and TNFα. The study was carried out with the use of Luminex equipment, and the results were analyzed using Luminex xPONENT acquisition software, version Analyst 5.1. In addition, blood plasma levels of C-reactive protein (CRP; Abcam), haptoglobin (Abcam), TGF-ß, prostaglandin E2 (PGE2) and IL-10 (R&D Systems), and IL-4 and porcine alpha-1 acid glycoprotein (AssayGenie) were quantified by ELISA according to manufacturer instructions.

#### Histopathological studies

During the third surgery, samples measuring approximately 5 mm^3^ were obtained of the spleen, kidney, small intestine, peritoneum, and cecum. These were then fixed in 10% formaldehyde at room temperature, embedded in paraffin, and cut into 5 micron-thick slices using a Microm HM 360 microtome. Sections were stained with hematoxylin–eosin to study the grade and location of the inflammatory infiltrate. All sections were examined by the same researcher, who was blinded to the experimental groups.

## Results

### Mortality

All peri- and postoperative procedures were uneventful and performed by the same surgical team. There were two deaths in the control group, 48 h after the first surgery, and one in the low-dose treatment group 96 h after the first intervention. There were no deaths in the high-dose treatment group. This gives a mortality rate of 15%, which is similar to that reported in the literature.

### Macroscopic findings

**Fig. 2:**
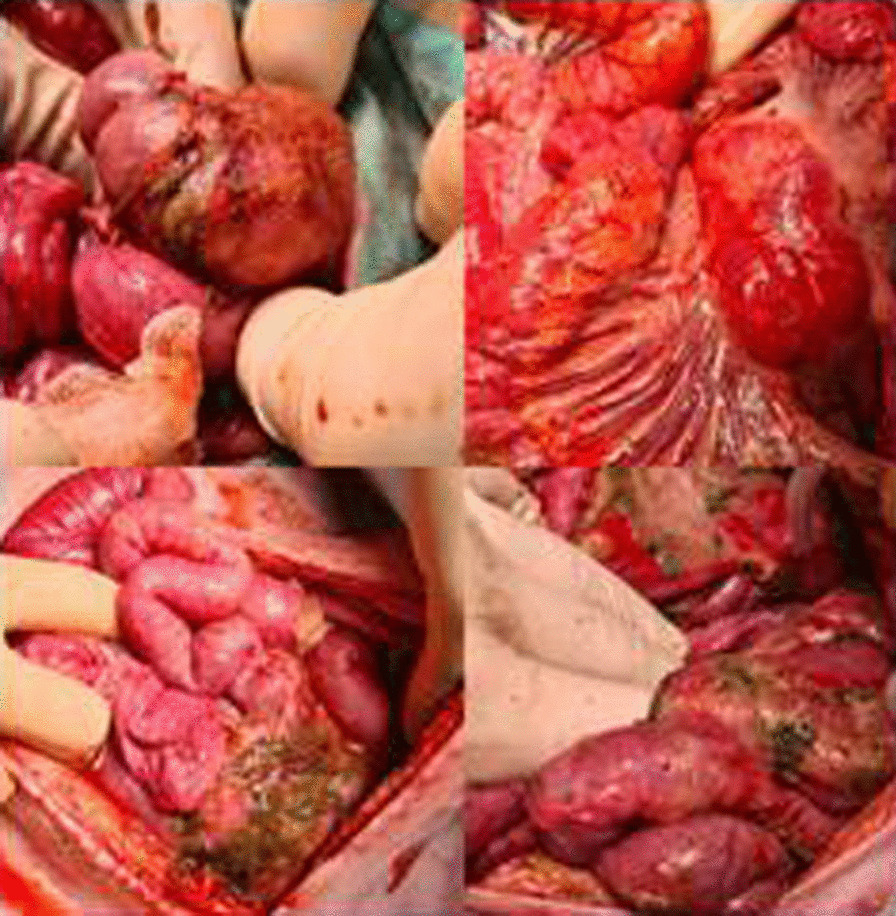
Finding 24 h after first surgery. Findings 24 h after the first surgery. Prior to lavage and treatment, signs of peritonitis are evident

Twenty-four hours after the first surgery, in which peritonitis was induced, all animals developed clinical signs of severe abdominal infection. Decreased activity, somnolence, tachypnea, tachycardia, and fever (temperature > 39.4 ± 0.5 °C) were present in all animals. Macroscopic findings were also similar in all animals following laparotomy. All animals presented diffuse fecal peritonitis, the most affected area being the left inferior quadrant; ascites in varying degrees; fibrin sheath formation; loose adhesions on the intestine and abdominal walls; adynamic ileus; and mesenteric adenitis. None of the animals showed suture dehiscence (Fig. 2).

**Fig. 3:**
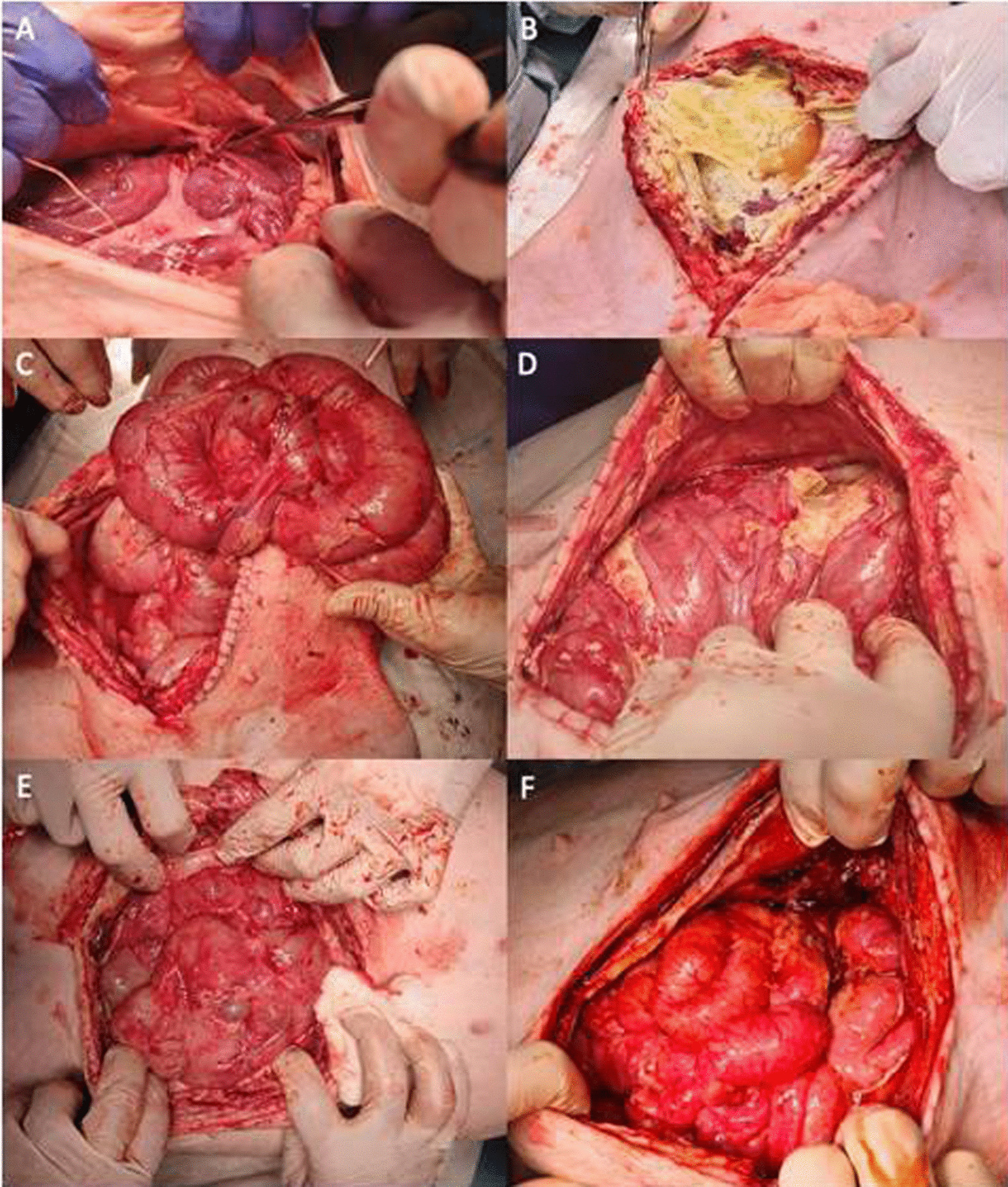
Findings at killing in the different groups. **A** and **B** Control group. **C** and **D** 2 × 10^6^ ADSC treatment group. **E** and **F** 4 × 10^6^ ADSC treatment group

At killing, animals in the control group showed intense fibrosis with dense intestinal adhesions and abdominal wall adhesions that were unable to be separated bluntly, moderate amounts of ascites, and, in some animals, interloop abscesses (Fig. 3A, B). Animals included in the 2 × 10^6^ ADSC treatment group showed less fibrosis compared to the control group. We observed somewhat firm interloop and wall adhesions, although these were more easily separated bluntly. A moderate volume of ascites was also present in some animals, and no abdominal abscesses were seen (Fig. 3C, D). Findings in the 4 × 10^6^ ADSC treatment group included a reduced level of fibrosis compared to the control group and the 2 × 10^6^ ADSC treatment group; loose interloop and abdominal wall adhesions that were easily separated with blunt dissection; a moderate volume of ascites in some animals; and no evidence of abdominal abscesses (Fig. 3E, F). Data contained in Additional file 3: Table S3 confirm difference between the group without cell treatment and the groups with different doses of cell treatment, but we did not observe any differences between the two cell groups.

### Blood test

Blood test results (biochemical and hematologic) were similar between groups, and no significant differences were observed (Additional file 1: Table S1). However, the neutrophil/lymphocyte count ratio in all groups increased considerably 24 h after the first surgery (mean ± SD cells groups 1.34 ± 0.3 vs. control 3.045 ± 1.2). Followed by a more rapid decrease in cell-treated groups compared to the control group at 48 h (1.16 ± 004 vs. 2.95 ± 1.2). Also, when tested at day 7 postoperatively, NLCR counts were lower in both treatment groups compared to the control group (0.81 ± 0.1 vs. 2.12 ± 1.0) (Additional file 2: Table S2) and we observed this difference associated with cell treatment. Slightly lower levels of fibrinogen were also seen in both treatment groups at 7 days. The control group had a higher platelet count, although all three groups were within the normal range.

We observed no differences between groups in partial thromboplastin time, prothrombin time, INR, CRP, haptoglobin, ferritin, fibrinogen, glucose, creatinine, bilirubin, ALT, GGT, or LDH. Using specific ELISA parameters, we analyzed the levels of porcine CRP, haptoglobin, IL-4, IL-10, PGE2, TGF-ß and porcine alpha-1 acid glycoprotein, as we considered these to be fundamental sepsis biomarkers according to existing clinical research. The results showed a significant increase of CRP and haptoglobin in all animals after the first surgical procedure.

**Fig. 4:**
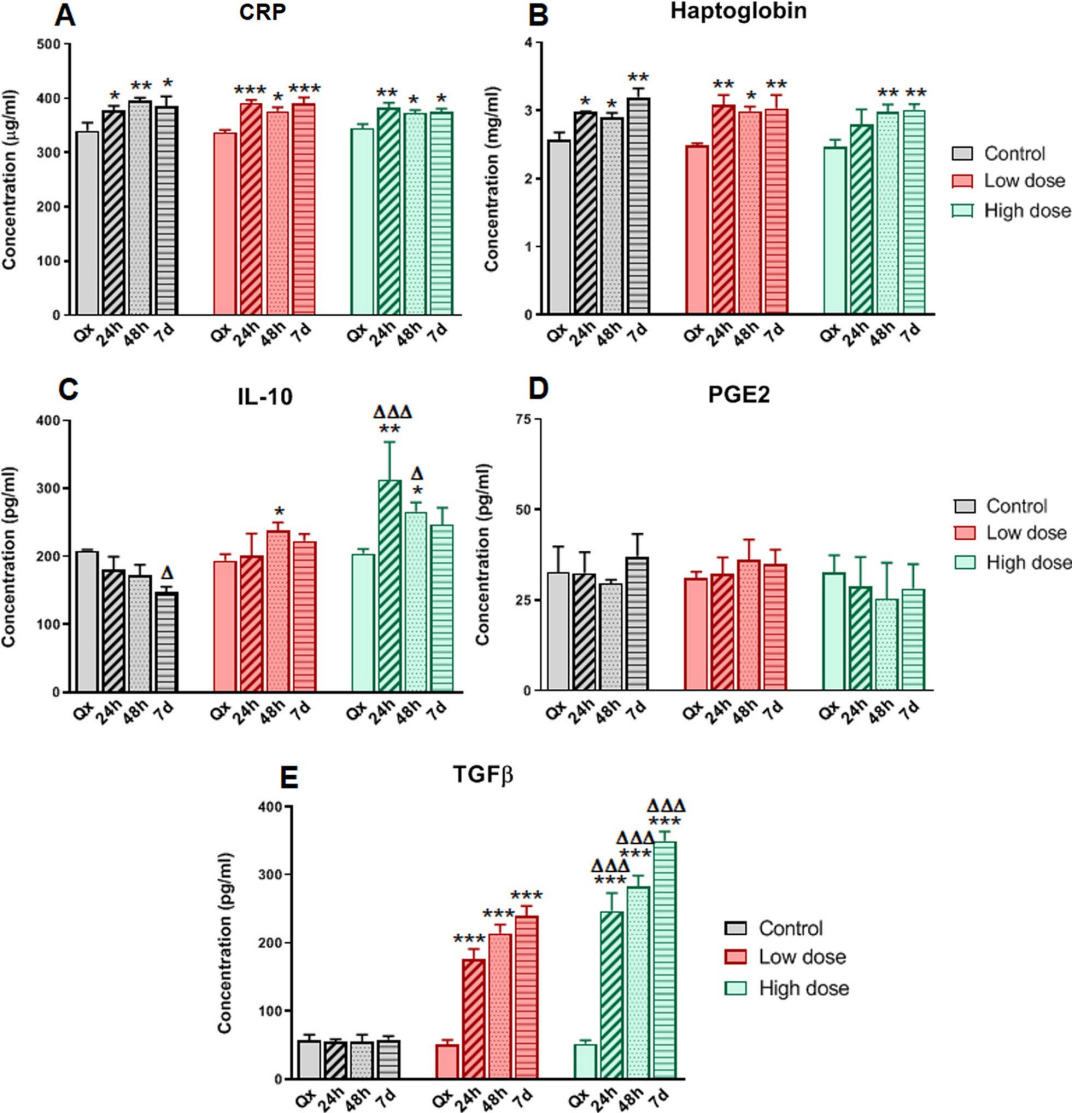
Specific ELISA of the more relevant parameters in peritoneal sepsis. Levels of each molecule were significantly increased compared to the control group (untreated) *p < 0.05, **p < 0.01, and ***p < 0.001, respectively, or significantly increased in the high cell dose compared to the low dose group *p < 0.05, and ***p < 0.001, respectively. Qx: animals undergoing surgery but without sepsis induction

Most importantly, there was a significant increase of IL-10 and TGF-ß in both ADSC treatment groups 24 h after the second surgery (Fig. 4). Unfortunately, IL-4 and alfa-1-acid glycoprotein levels were not detectable.

As expected, microbiological blood cultures were positive, revealing presence of microbial flora of the gastrointestinal tract (mainly *Escherichia coli*, *Klebsiella pneumoniae*, and *Streptococcus suis*). All animals in the low-dose ADSC treatment group except one had at least one positive culture after peritonitis induction surgery.

### Blood cytokine analysis

The results of blood cytokine levels at different time points are shown in Table 2. A highly significant increase in IL-1Ra, IL-1b, IL-6, IL-8, TNF-α, and IL-10 was observed 24 h after induction of abdominal infection in all animals (at this time point, all groups had similar values). IFNγ and IL-12 concentrations showed a slight decrease over the first 24 h.

**Table 2:** Results of cytokine analysis in peritoneal lavage (mean)

Analyte sample	GM-CSF	IFNg	IL-1a	IL-1Ra	IL-1b	IL-2	IL-6	IL-8	IL-10	IL-12	IL-18	TNF-α
	ng/mL	ng/mL	ng/mL	ng/mL	ng/mL	ng/mL	ng/mL	ng/mL	ng/mL	ng/mL	ng/mL	ng/mL
Pre-surgery	0	0.69	0.22	**0.46**	**0.08**	0.01	**0.04**	**0.02**	0.02	0.23	0.91	0.02
24 h post-surgery	0.02	0.04	0.30	**6.21**	**15.5**	0.01	**1.45**	**1.78**	0.07	0.05	0	0.04

The most relevant variations are bold

At 48 h after initial surgery (i.e., 24 h post-treatment), the treatment groups showed a significant decrease (*p* < 0.02) in IL-1Ra and IL6, although no such decrease was found in the control group. We also observed an increase in IL-1b, IL-12, and TNF-α in all three groups.

At day 7, levels of IL-1Ra, IL-2, and IL-6 were elevated in the control group, but only IL-2 was elevated in the ADSC-treated groups, and we observed a significant decrease in IFNγ between the two treatment groups: The high-dose group showed more increased or decreased values, although the differences did not reach statistical significance. The remaining cytokines had similar levels for all groups and time points. Importantly, while PGE2 levels did not change after induction of sepsis in the different groups (Fig. 3D), IL-10 (up to 48 h) and TGF-ß levels (up to 7 days) increased significantly in the groups receiving ADSC treatment, especially those receiving a high dose (Fig. 4C, E, Table 2).

### Peritoneal lavage

Peritoneal lavage was only analyzed 24 h after the induction of peritonitis. Samples were collected after 3000 cc of saline lavage had been administered to the peritoneal cavity to avoid high concentrations of fecal remnants and/or fibrotic tissue. The mean of the most relevant results of the cytokine analysis is shown in Table 3. Pro-inflammatory cytokines were elevated after 24 h of peritonitis formation, especially IL-1Ra, IL-1b, IL-6, IL-8, and TNF-α. IL-4 and IL-18 were not detectable.

**Table 3:** Cytokine analysis results in blood

Group	Analyte sample	GM-CSF	IFNg	IL-1a	IL-1Ra	IL-1b	IL-2	IL-6	IL-8	IL-10	IL-12	IL-18	TNF-α
		ng/mL	ng/mL	ng/mL	ng/mL	ng/mL	ng/mL	ng/mL	ng/mL	ng/mL	ng/mL	ng/mL	ng/mL
Control	Pre-surgery	0.015	0.043	0.005	0.42	0.078	0.013	0.015	0.007	0.086	0.802	0.1	0.012
	24 h post-S	0.015	0.038	0.008	33.21	0.084	0.024	1.573	0.01	0.279	0.751	0.25	0.063
	48 h post-S	0.015	0.038	0.007	10.46	0.7	0.016	0.223	0.007	0.133	0.351	0.2	0.033
	7 d post-S	0.015	0.038	0.015	4.45	0.54	0.036	0.174	0.007	0.165	0.345	0.1	0.022
Low dose	Pre-surgery	0.015	0.038	0.009	0.13	0.078	0.029	0.027	0.007	0.102	0.633	0.2	0.016
	24 h post-S	0.017	0.041	0.01	11.56	0.045	0.038	0.528	0.005	0.489	0.342	0.4	0.036
	48 h post-S	0.015	0.038	0.008	2.77	0.30	0.034	0.121	0.002	0.329	0.257	0.36	0.022
	7 d post-S	0.015	0.038	0.032	1.56	0.186	0.117	0.11	0.002	0.393	0.279	0.04	0.012
High dose	Pre-surgery	0.015	0.043	0.014	0.159	0.078	0.038	0.023	0.007	0.132	2.178	0.01	0.017
	24 h post-S	0.015	0.043	0.012	17.84	0.637	0.042	1.01	0.002	0.356	1.348	0.03	0.028
	48 h post-S	0.015	0.043	0.021	2.65	0.489	0.048	0.184	0.001	0.284	1.013	0.027	0.021
	7 d post-S	0.015	0.038	0.027	0.62	0.16	0.087	0.048	0.001	0.294	0.812	0.03	0.013

Mean of 3 assays, in all cases SD < 10%

### Histology

**Fig. 5:**
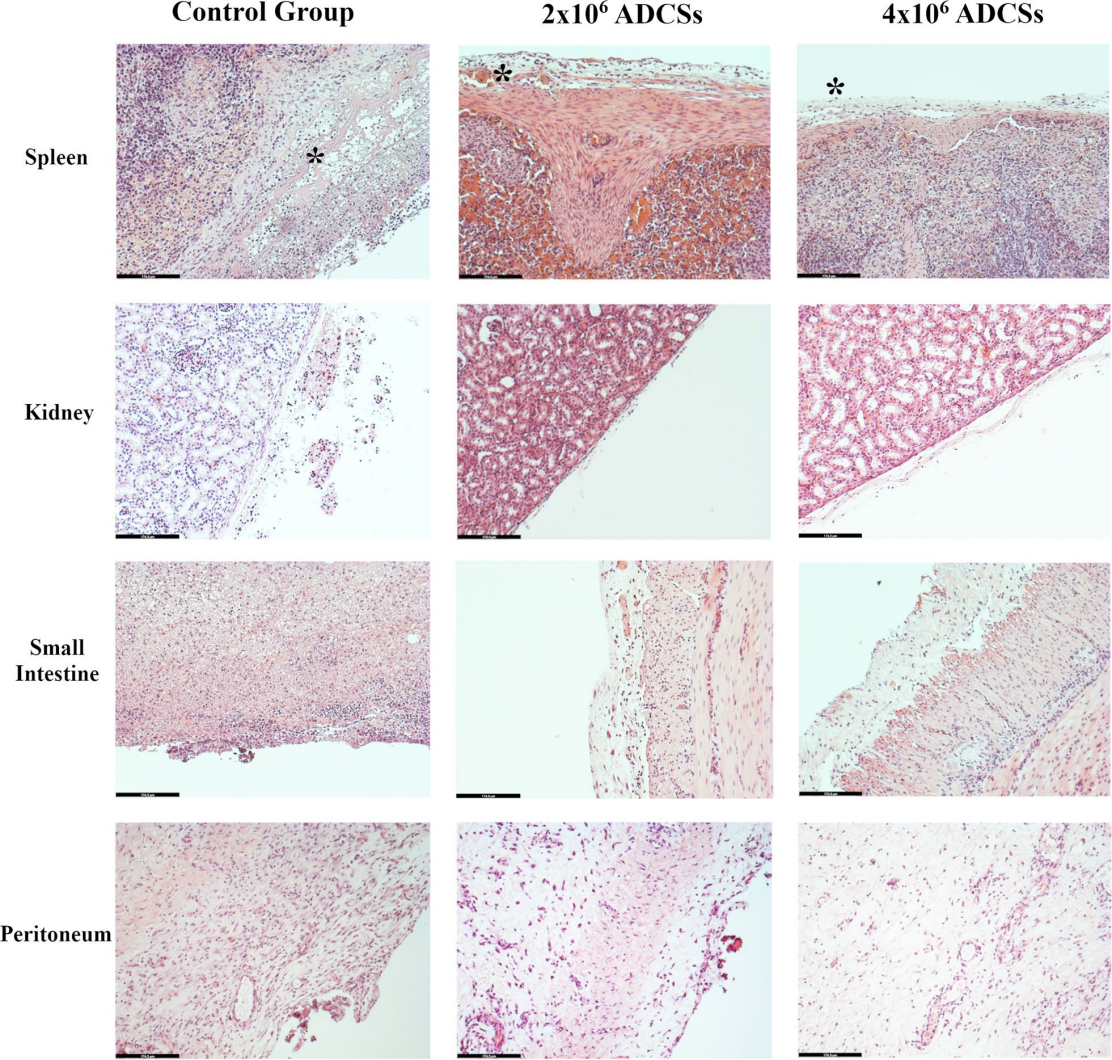
Histological analysis. Histological images of the different tissue samples from the three groups studied, stained with hematoxylin and eosin. Images were acquired at 100 × magnification at the most significant regions. Inflammatory infiltrate (asterisk) in the mesentery surrounding the spleen is in evidence, although the connective tissue capsule remains intact. Striking presence of inflammatory infiltrate in the serosa of pigs in the control group as compared to the highly reduced presence of infiltrate in animals treated with either low- or high-dose ADSCs. Evidence of the same pattern between treatment groups and controls in the peritoneum. No inflammatory involvement in kidney samples from any group

The small intestine, cecum, peritoneum, spleen, and kidney were analyzed histopathologically (Fig. 5). The infiltrate obtained was analyzed by means of neutrophil/plasma cell ratio. We carried out a quantitative analysis of the results obtained using a Pearson test. Differences were parametric, and comparisons were made using the ANOVA test and Student's t test for paired samples. Data are presented as mean ± the standard deviation of the mean (SD). In all cases, *p* values less than 0.05 were considered significant (Additional file 2: Table S2).

There was evidence of inflammatory infiltrate in the serosa of the small intestine and cecum in all groups studied, but this infiltrate was more intense in the control group than in the ADSC treated groups (*p* < 0.05). The high-dose ADSC treatment group had the least inflammatory infiltrate of all specimens.

In all animals studied, we found that the layers underlying the serosa had a preserved histologic structure and did not show signs of inflammatory infiltrate. In the peritoneum, inflammation was found only in the most superficial layer in control group (*p* < 0.05); in the spleen only the mesentery was affected mainly in control group (*p* < 0.05). In the kidney, no differences were observed between the study groups.

## Discussion

Several studies have evaluated the efficacy of MSCs in treating sepsis, and a recent metaanalysis showed that MSC therapy for sepsis reduced mortality in preclinical studies [12]. Most existing preclinical studies were performed in rodents and translate poorly to human patients. This study on the effect of ADSC treatment in sepsis uses a larger mammal to create an animal model that simulates an abdominal perforation/anastomotic leak, thereby allowing analysis of time to detection and treatment as closely as possible. The modifications made to the sepsis model proposed by Kubiak et al. [18] included closure of the enterotomy to ensure equal contamination of all subjects, elimination of clamping of the superior mesenteric artery to eliminate ischemia/reperfusion injury, and a reduction in the amount of blood in the clot formation to facilitate lavage. In contrast to the model proposed by Horak et al. [17], we used xenogeneic adipose-derived MSCs and an innovative intraperitoneal delivery. This model conforms to the Minimum Quality Threshold in Pre-Clinical Sepsis Studies (MQTIPSS) [19]. Our results demonstrate that the protocol is feasible, and microbiological findings show that we have produced peritonitis and bacteremia in all cases, with a mortality rate similar to that observed in previous publications.

Earlier studies have shown an improvement in fibrosis in various diseases when treated with MSC therapy [20], and other research has reported a reduction in adhesions with intravenous MSC treatment [21–23]. When MSCs are applied intraperitoneally, results are inconsistent, with some showing no benefit [21, 24], and others showing benefit [25, 26].

The mechanisms responsible for the reduction in adhesions are not clear. Some authors suggest that MSCs differentiate into mesothelial cells and repair damaged peritoneum [25], while others point to the anti-inflammatory and immunomodulatory properties of these stem cells [27]. In our study, although macroscopic changes varied from animal to animal, an overall improvement was seen with ADSC treatment, as the amount and consistency of adhesions was reduced, making them looser and easier to blunt dissect. In terms of mortality, even though there were fewer deaths in the ADSC group, the difference did not reach statistical significance due to the small number of subjects in each group.

One noteworthy finding is the clinical improvement observed after ADSC treatment. Animals in the treatment groups had increased mobility and food intake and decreased body temperature 48 h after infusion. In contrast, the animals in the control group were apathetic, had difficulty moving, ate less, and their body temperature did not decrease after sepsis induction.

Unfortunately we were unsuccessful in our attempt to locate human cells in the different organs of the peritoneal cavity after 7 days of cell administration. We believe that this must be related to the dispersion of the cells in the peritoneal cavity. In previous studies in rats or mice, we found a small number of cells throughout the life of the rodent [28].

Complete blood count is a fundamental tool for monitoring clinical response to infection. Parameters such as mean platelet volume (MPV), red cell distribution width (RDW), and the neutrophil/lymphocyte count ratio (NLCR) have been shown to be diagnostic and prognostic factors [27, 29]. Neutrophil/lymphocyte count ratio (NLCR) is a simple and inexpensive biomarker and can be used in sepsis as a diagnostic and prognostic tool [30]. Counts are obtained by dividing the number of neutrophils by the number of lymphocytes. As a diagnostic marker, it has a sensitivity of 87.5% and a specificity of 90% at a value of > 3.3. When used for prognosis a high NLCR on day 5 is associated with a poor outcome with a sensitivity of 73% and a specificity of 71%. [31]. We observed an increase in NLCR 24 h after the first surgery in all cases, suggesting an acute inflammatory process. The fact that leukocytes decreased more rapidly to near normal levels in both ADSC treatment groups suggests a reduction in inflammation [32] (Additional file 3: Table S3). The lower presence of neutrophils in the histological samples from the two treatment groups also suggests the potential of these treatments to inhibit fibrosis, thereby facilitating resolution of sepsis [33].

Twenty-four hours after the first intervention, peritoneal lavage fluid showed an increase in several interleukins, mainly IL-1Ra and IL1a (13.5- and 194-fold increase, respectively), associated with high fever and positive blood cultures in all animals studied, thus indicating the development of peritonitis with bacteriemia. Intestinal dysbiosis has been associated with elevated levels of IL-1a [34] and a response to infection generated by IL-1Ra causing fever, neutrophilia, and acute phase protein production [35].

In addition to the higher levels of CRP and haptoglobin observed, we found a very significant increase in IL-1Ra, IL-6, and IL-8 in the blood of all animals 24 h after sepsis onset. Most of these are pro-inflammatory cytokines that activate the immune response against infection. The slight increase in IL-1a, IL-10, and TNF-α, together with the decrease in IL-12 and IFNγ, indicates that the immune process in the blood undergoes a physiological evolution. The increase further reveals that at 24 h the immune response has been triggered and is part of the initial host response to injury, which is responsible for some of the clinical features of infection and systemic inflammatory response syndrome (SIRS) [36]. These data, together with the significant increase in IL-1Ra, signal that tissue and organ damage has begun, as seen in previous studies [37, 38]. At 24 h post-treatment (48 h after the first surgery), the levels of interleukins in the control group were maintained compared to the previous measurements, indicating that the animals remained prone to fibrosis and multiorgan failure despite antibiotic treatment. In the groups treated with MSCs, on the other hand, a slight decrease in proinflammatory cytokines was observed, accompanied by an increase in TNF-α, IL-10, and a decrease in IL1Ra and IL-12 and, especially in the group with the lowest cell dose, suggesting a change from a pro-inflammatory to an anti-inflammatory response [35–37]. Finally, 7 days after the induction of peritoneal sepsis, the animals in the control group continued to show an increase in inflammatory mediators such as IL-1Ra, IL-6, and IL-2. In contrast, the cell-treated groups had significantly reduced levels of IFN, while maintaining high levels of TNF-α and significantly high levels of IL-2. The mechanism by which these mediators favor the resolution of the infectious/inflammatory process is still unclear, although it seems that it may be associated with regulatory T cell activation [39], or by activating the innate response through IFNs by increasing macrophage and natural killer (NK) cell generation [40], or maintaining macrophage phagocytic activity (as observed by the sustained levels of TNF-α) [41], or even by the intrinsic antimicrobial properties of MSCs (as observed by the production of antimicrobial peptides such as hCAP-18/LL-37) [42].

IL-2 plays a key role in immunity and tolerance, increasing the cytolytic activity of CD8 + and NK lymphocytes. IL-2 also regulates naïve lymphocytes toward Th1 and Th2 differentiation. Due to all these functions, IL-2 is an important initiator of cellular immunity [43–45]. At 48 h and 7 days of treatment, levels of this cytokine in the ADSC treatment groups were more than double those of the control group, which indicates a more robust and faster transition to adaptive immunity.

Consistent with the data obtained in multiplex assays, when we performed specific ELISA against porcine PCR we observed an increase in all cases, thus mirroring the clinical evolution in humans. Furthermore, the most relevant findings were seen with stem-cell treatment, where a significant increase of IL-10 was observed in blood. These data, together with the increase in TGF-ß, suggest a potent anti-inflammatory effect of ADSCs. Although the specific mechanisms that make this possible are unclear, our data suggest a regulation of the immune system toward an increase in type II macrophages, thus generating an anti-inflammatory profile that aids in resolving sepsis [46]. Another mechanism of ADSCs that contributes to the control of inflammatory conditions is the promotion of T regulatory cell (Tregs) differentiation. The increase in IL-10 and TFG-ß may indicate that ADSCs activate Tregs by cell contact, increasing circulating levels of Tregs during sepsis [47].

A key aspect of our study concerns the xenogeneic use of MSC. Our results show that no significant immune response was triggered, thereby supporting the hypothesis of Hoogduijn and Lombardo [42] that ADSCs are immune-privileged cells. Although their mechanisms of action remain unknown, our results suggest that the use of MSC xeno-transplants is not sufficient to generate a clinically relevant immune response and also indicate that ADSCs behave as immunoregulators, favoring the resolution of inflammation and the inflammatory imbalance generated by peritoneal sepsis [48–51].

Our results are consistent with most published preclinical studies, as we found that MSC therapy improves outcomes in a sepsis model. As mentioned above, however, this previous research is mainly based on small animals [12]. To our knowledge, only 2 studies have evaluated the effect of MSCs in a porcine model of sepsis. Laroye et al., who used umbilical cord-derived MSCs, reported improved hemodynamic parameters and survival in a porcine model of septic shock [49]. In contrast, Horak et al. found no treatment benefit [17]. In both studies, follow-up duration was less than 24 h until killing, no antibiotic treatment was initiated, and no source control was performed.

Due to the complex clinical course of sepsis, the varying dose levels and routes of administration used, as well as the heterogeneity of MSC extraction protocols, caution should be exercised when interpreting the results of preclinical studies in animal models of sepsis and treatment with MSCs.

In this study, we have analyzed the effects of two different cell doses during treatment. We understand that the number of animals per group does not allow us to arrive at definitive conclusions, but if we combine the results of both groups it seems that the highest dose produces less abdominal fibrosis, an increase in IL-10 and TGF-β in the blood and a lower inflammatory infiltrate in tissues. However, the differences are not significant and in our opinion if clinical value is the aim, the cost of greater production would make this step more complex.

Despite the limitations of the present study, such as its open-label design and lack of invasive monitoring, certain strengths make the methods and results relevant. Firstly, the animal model more closely resembles routine clinical practice in terms of time to diagnosis and treatment. Secondly, ADSC treatment was evaluated as an adjuvant to routine management and not as an alternative approach. Finally, the use of human xenogeneic cells leads us to believe that the results may be more readily transferable to clinical practice, especially considering that the follow-up time is much longer than previous animal studies and more closely resembles the clinical course of the disease.

## Conclusions

Intraperitoneal administration of adipose-derived mesenchymal stem cells as an adjunct to standard treatment is safe and feasible, improves the outcome, and reduces intra-abdominal adhesions in a clinically relevant porcine model of abdominal sepsis. Despite these promising results, continued preclinical research is needed before the approach can be translated to clinical practice due to the complexity of the clinical course of sepsis and variability in the dose and route of administration of the proposed treatment.

### Supplementary Information

**Additional file 1. Table S1. **Hematological and biochemical analysis.

**Additional file 2. TableS2. **Histological analysis of inflammatory cells.

**Additional file 3. TableS3. **Macroscopic findings and Neutrophil/lymphocyte count ratio (NLR).

## Data Availability

The datasets analyzed during the current study available from the corresponding author. All data generated during this study are included in this published article [and its Additional files].

## Abbreviations

ADSC: Adipose-derived mesenchymal stem cells
ALT: Alanine transaminase
ßFGF: Basic fibroblast growth factor
CRP: C-reactive protein
GGT: Gamma-glutamyl transferase
IFNγ: Interferon gamma
LDH: Lactate dehydrogenase
MHC-I: Major histocompatibility complex class I
MPV: Mean platelet volume
MQTIPSS: Minimum quality threshold in pre-clinical sepsis studies
MSC: Mesenchymal stem cells
NK: Natural killer cells
NLCR: Neutrophil/Lymphocyte count ratio
PGE2: Prostaglandin E2
RDW: Red cell distribution width
SIRS: Systemic inflammatory response syndrome
SOFA: Sequential Organ Failure Assessment scale
TGF ß: Transforming growth factor beta
TNF-α: Tumor necrosis factor alfa
Tregs: T regulatory cells
