# Supplementary material for: Therapeutic effect of adipose-derived mesenchymal stem cells in a porcine model of abdominal sepsis
Source: Stem Cell Res Ther. 2023 Dec 12;14:365. doi: 10.1186/s13287-023-03588-x (PMC10717819; doi:10.1186/s13287-023-03588-x)
Supplement: Supplementary file 1 — Additional file 1. Table S1. Hematological and biochemical analysis. [file 13287_2023_3588_MOESM1_ESM.docx]

|  | | **CONTROLS** | | | | | | | | | | | | | | | | | | | | |  |
| --- | --- | --- | --- | --- | --- | --- | --- | --- | --- | --- | --- | --- | --- | --- | --- | --- | --- | --- | --- | --- | --- | --- | --- |
|  | |  |  |  |  |  |  |  |  |  |  |  |  |  |  |  |  |  |  |  |  |  |  |
|  | | **Qx** | | | | **24h** | | | | | | **48h** | | | | | **7d** | | | | | |  |
|  | | Average | | SD | | Average | | | SD | | | Average | | SD | | | Average | | | SD | | |  |
| **Leukocytes** | | 16456.67 | | 7195.57 | | 15168.71 | | | 9229.53 | | | 21140.00 | | 4669.95 | | | 35966.00 | | | 11806.97 | | |  |
| **Red blood cells** | | 5816666.67 | | 420317.34 | | 7285714.29 | | | 829515.06 | | | 5783333.33 | | 343025.75 | | | 6700000.00 | | | 1806931.10 | | |  |
| **Haemoglobin** | | 9.15 | | 1.38 | | 11.56 | | | 1.92 | | | 9.05 | | 1.39 | | | 10.70 | | | 2.87 | | |  |
| **Haematocrit** | | 32.55 | | 4.26 | | 40.61 | | | 6.33 | | | 31.42 | | 4.03 | | | 37.44 | | | 10.68 | | |  |
| **Platelets** | | 567833.33 | | 139989.17 | | 479714.29 | | | 118267.65 | | | 387333.33 | | 166537.28 | | | 601400.00 | | | 158241.27 | | |  |
| **% lymphocytes** | | 54.98 | | 8.14 | | 32.77 | | | 9.38 | | | 33.12 | | 3.39 | | | 46.68 | | | 10.38 | | |  |
| **% monocytes** | | 7.17 | | 3.32 | | 6.15 | | | 2.34 | | | 6.40 | | 2.48 | | | 4.14 | | | 1.24 | | |  |
| **% segmented** | | 36.57 | | 10.38 | | 60.80 | | | 9.15 | | | 58.67 | | 5.86 | | | 48.67 | | | 10.05 | | |  |
| **Lymphocytes (ABS)** | | 8666.67 | | 3116.84 | | 5328.57 | | | 1832.77 | | | 7133.33 | | 1875.81 | | | 16380.00 | | | 4937.31 | | |  |
| **Monocytes (ABS)** | | 1050.00 | | 398.75 | | 1275.00 | | | 693.85 | | | 1200.00 | | 376.39 | | | 1540.00 | | | 832.47 | | |  |
| **Segmented (ABS)** | | 6583.33 | | 4196.86 | | 10714.29 | | | 5364.21 | | | 12566.67 | | 3008.43 | | | 21166.67 | | | 9012.60 | | |  |
| **Eosinophils (ABS)** | | 116.67 | | 98.32 | | 57.14 | | | 78.68 | | | 333.33 | | 273.25 | | | 260.00 | | | 343.51 | | |  |
| **Basophils (ABS)** | | 66.67 | | 57.74 | | 0.00 | | | 0.00 | | | 33.33 | | 51.64 | | | 100.00 | | | 100.00 | | |  |
| **Fibrinogen derived** | | 672.60 | | 53.32 | | 1319.83 | | | 138.95 | | | 1328.29 | | 171.90 | | | 805.33 | | | 221.25 | | |  |
| **Alkaline phosphatase** | | 352.80 | | 120.18 | | 368.40 | | | 84.48 | | | 222.33 | | 42.83 | | | 94.00 | | | 6.24 | | |  |
| **LDH** | | 618.33 | | 113.15 | | 728.33 | | | 174.02 | | | 883.14 | | 161.15 | | | 605.50 | | | 174.82 | | |  |
|  | **Low-dose ADSC** | | | | | | | | | | | | | | | | | | | | | | |
|  |  |  |  |  |  |  |  |  |  |  |  |  |  |  |  |  |  |  |  |  |  |  |  |
|  | **Qx** | | | | | | **24h** | | | | | | **48h** | | | | | | **7d** | | | | |
|  | Average | | | | SD | | Average | | | | SD | | Average | | | SD | | | Average | | SD | | |
| **Leukocytes** | 16156.67 | | | | 8329.23 | | 11930.00 | | | | 8658.04 | | 18370.00 | | | 5083.62 | | | 25296.00 | | 14763.39 | | |
| **Red blood cells** | 5397777.78 | | | | 1965717.2 | | 6077777.7 | | | | 736667.2 | | 5320000.0 | | | 334664.0 | | | 6114285.7 | | 380788.66 | | |
| **Haemoglobin** | 9.86 | | | | 1.34 | | 10.44 | | | | 1.36 | | 8.54 | | | 0.71 | | | 9.94 | | 0.63 | | |
| **Haematocrit** | 36.41 | | | | 4.88 | | 41.06 | | | | 5.06 | | 32.26 | | | 3.07 | | | 36.48 | | 2.48 | | |
| **Platelets** | 438111.11 | | | | 141073.96 | | 352750.00 | | | | 150717.1 | | 370200.00 | | | 162282.78 | | | 508800.00 | | 167790.64 | | |
| **% lymphocytes** | 60.66 | | | | 9.66 | | 40.51 | | | | 14.73 | | 40.38 | | | 13.49 | | | 54.04 | | 16.14 | | |
| **% monocytes** | 4.89 | | | | 2.16 | | 5.28 | | | | 3.71 | | 10.56 | | | 5.72 | | | 2.42 | | 0.93 | | |
| **% segmented** | 33.78 | | | | 8.76 | | 53.60 | | | | 16.07 | | 47.86 | | | 16.81 | | | 42.58 | | 15.70 | | |
| **Lymphocytes (ABS)** | 9533.33 | | | | 3591.31 | | 4437.50 | | | | 3138.67 | | 7180.00 | | | 2088.54 | | | 12700.00 | | 6968.86 | | |
| **Monocytes (ABS)** | 866.67 | | | | 672.68 | | 487.50 | | | | 203.10 | | 1280.00 | | | 708.52 | | | 640.00 | | 433.59 | | |
| **Segmented (ABS)** | 6188.89 | | | | 4610.98 | | 6950.00 | | | | 5923.32 | | 9180.00 | | | 4964.57 | | | 11740.00 | | 10025.37 | | |
| **Eosinophils (ABS)** | 127.81 | | | | 100.12 | | 116.14 | | | | 63.63 | | 89.91 | | | 36.28 | | | 91.10 | | 27.89 | | |
| **Basophils (ABS)** | 78.97 | | | | 59.23 | | 41.15 | | | | 22.06 | | 29.48 | | | 7.99 | | | 77.14 | | 41.33 | | |
| **Fibrinogen derived** | 776.25 | | | | 266.55 | | 1316.56 | | | | 199.02 | | 1339.17 | | | 150.38 | | | 684.33 | | 110.62 | | |
| **Alkaline phosphatase** | 292.44 | | | | 123.41 | | 374.88 | | | | 105.30 | | 266.20 | | | 43.24 | | | 338.80 | | 207.74 | | |
| **LDH** | 662.88 | | | | 94.02 | | 664.71 | | | | 138.33 | | 865.00 | | | 34.42 | | | 788.80 | | 102.51 | | |
|  | **High-dose ADSC** | | | | | | | | | | | | | | | | | | | | | |  |
|  |  |  |  |  |  |  |  |  |  |  |  |  |  |  |  |  |  |  |  |  |  |  |  |
|  | **Qx** | | | | | | | **24h** | | | | | **48h** | | | | | **7d** | | | | |  |
|  | Average | | SD | | | | | Average | | SD | | | Average | | SD | | | Average | | | | SD |  |
| **Leukocytes** | 15110 | | 3054.70 | | | | | 19505 | | 1944.54 | | | 22880 | | 4737.62 | | | 23960 | | | | 3761.81 |  |
| **Red blood cells** | 5600000 | | 0.00 | | | | | 5750000 | | 919238.82 | | | 5850000 | | 353553.39 | | | 5950000 | | | | 70710.68 |  |
| **Haemoglobin** | 9.4 | | 0.57 | | | | | 9.6 | | 0.99 | | | 9.6 | | 0.28 | | | 9.6 | | | | 0.14 |  |
| **Haematocrit** | 33 | | 2.26 | | | | | 33.9 | | 3.11 | | | 35.1 | | 0.14 | | | 34.05 | | | | 0.21 |  |
| **Platelets** | 552000 | | 59396.97 | | | | | 457000 | | 48083.26 | | | 435000 | | 65053.82 | | | 408000 | | | | 19798.99 |  |
| **% lymphocytes** | 64 | | 3.25 | | | | | 29.75 | | 14.21 | | | 35.75 | | 17.47 | | | 42.55 | | | | 16.62 |  |
| **% monocytes** | 6.05 | | 3.89 | | | | | 3.5 | | 0.14 | | | 5.65 | | 1.91 | | | 2.9 | | | | 0.99 |  |
| **% segmented** | 28.55 | | 7.57 | | | | | 65.5 | | 15.70 | | | 56.15 | | 21.57 | | | 52.55 | | | | 18.17 |  |
| **Lymphocytes (ABS)** | 9650 | | 1484.92 | | | | | 5650 | | 2192.03 | | | 7750 | | 2333.45 | | | 9900 | | | | 2404.16 |  |
| **Monocytes (ABS)** | 850 | | 353.55 | | | | | 700 | | 0.00 | | | 1250 | | 212.13 | | | 700 | | | | 141.42 |  |
| **Segmented (ABS)** | 4400 | | 1979.90 | | | | | 12950 | | 4313.35 | | | 13400 | | 7636.75 | | | 12950 | | | | 6293.25 |  |
| **Eosinophils (ABS)** | 132.18 | | 96.20 | | | | | 121.14 | | 67.01 | | | 94.16 | | 41.07 | | | 99.68 | | | | 31.60 |  |
| **Basophils (ABS)** | 67.17 | | 42.92 | | | | | 38.34 | | 19.55 | | | 32.06 | | 11.00 | | | 66.93 | | | | 29.91 |  |
| **Fibrinogen derived** | 693.5 | | 118.09 | | | | | 1490 | | 377.95 | | | 1237.67 | | 196.51 | | | 707.67 | | | | 168.53 |  |
| **Alkaline phosphatase** | 180.33 | | 50.21 | | | | | 312.67 | | 37.58 | | | 221 | | 8.89 | | | 104.33 | | | | 31.97 |  |
| **LDH** | 601.67 | | 28.68 | | | | | 582 | | 80.99 | | | 690 | | 151.62 | | | 552.67 | | | | 66.20 |  |

**Supplementary TABLE 1**. Hematological and biochemical analysis

T
