# Supplementary material for: Therapeutic effect of adipose-derived mesenchymal stem cells in a porcine model of abdominal sepsis
Source: Stem Cell Res Ther. 2023 Dec 12;14:365. doi: 10.1186/s13287-023-03588-x (PMC10717819; doi:10.1186/s13287-023-03588-x)
Supplement: Supplementary file 2 — Additional file 2. TableS2. Histological analysis of inflammatory cells. [file 13287_2023_3588_MOESM2_ESM.docx]

Supplementary Table 2. Histological analysis of inflammatory cells

|  | **Neutrophils (±SD cells x field 400x)** | | | **Plasma cells (±SD cells x field 400x)** | | |
| --- | --- | --- | --- | --- | --- | --- |
|  | **Control Group** | **Low- doses group** | **High-doses group** | **Control Group** | **Low- doses group** | **High-doses group** |
| *SPLEEN******** | 23.1± 10.4 | 15.4±7.9 | 12.5±6.1 | 13.4±4.2 | 23.9±5.4 | 20.9±6.3 |
| *SMALL INTESTINE******** | 22.9±8.4 | 13.3±7.9 | 10.6±4.3 | 12.9±3.7 | 25.7±6.7 | 23.2±5.6 |
| *PERITONEUM******** | 24.1±6.6 | 16.9±7.7 | 11.7±5.1 | 13.1±4.1 | 23.5±5.9 | 24.1±5.8 |
| *KIDNEY* | 11.4±4.9 | 1.7±2.2 | 3.1±3.2 | 10.7±3.6 | 1.4±1.6 | 2.1±1.9 |

The infiltrate obtained was analysed with a neutrophil/plasma cell ratio. ****P ≤0.05.***
