# Supplementary material for: Therapeutic effect of adipose-derived mesenchymal stem cells in a porcine model of abdominal sepsis
Source: Stem Cell Res Ther. 2023 Dec 12;14:365. doi: 10.1186/s13287-023-03588-x (PMC10717819; doi:10.1186/s13287-023-03588-x)
Supplement: Supplementary file 3 — Additional file 3. TableS3. Macroscopic findings and Neutrophil/lymphocyte count ratio (NLR). [file 13287_2023_3588_MOESM3_ESM.docx]

Supplementary Table 3. Macroscopic findings and Neutrophil/lymphocyte count ratio (NLR)

|  |  |  |  | **NLR** | | |
| --- | --- | --- | --- | --- | --- | --- |
|  | **Abscesses ()** | **Ascites (mL)** | **Adherences** | **24h (±SD)** | **48h(±SD)** | **7d(±SD)** |
| **Control** | 1 | 1000±102 | firm | 3.045±1.21 | 2.95±1.20 | 2.12±1.06 |
| **Low-Dose** | 0.3* | 500±80 | lax | 1.47±0.61 | 1.16±0.06 | 0.80±0.10 |
| **High-Dose** | 0 | 250±20 | lax | 1.35±0.06 | 1.16±0.01 | 0.83±0.12 |

* In 7 animals without abcesses and 3 animals 1 abcesse
